# Surgical Treatment and Rehabilitation of Bilateral Popliteal Artery Entrapment Syndrome in a Young Boxer: A Case Report

**DOI:** 10.7759/cureus.49199

**Published:** 2023-11-21

**Authors:** Naosuke Nagata, Hiroshi Sasaki, Fumimasa Maruno

**Affiliations:** 1 Sports Medicine, Kobe Rosai Hospital, Kobe, JPN; 2 Orthopedic Surgery, Kobe Rosai Hospital, Kobe, JPN

**Keywords:** rehabilitation, treatment, intermittent claudication, young athletes, popliteal artery entrapment syndrome

## Abstract

Popliteal artery entrapment syndrome, a rare vascular disease observed in young athletes, is characterized by intermittent claudication and is often overlooked by orthopedists. Popliteal artery entrapment syndrome should be treated promptly when diagnosed, as the vascular lesion can progress.

We present a case of bilateral popliteal artery entrapment syndrome in a young professional boxer with no significant family or past medical history. He had developed intermittent claudication during a boxing match with pain in both calves, making it impossible for him to continue for more than three rounds. He was diagnosed with popliteal artery entrapment syndrome, and surgery treatment with reconstruction of the medial gastrocnemius muscle to maintain muscle strength was performed in collaboration with a cardiovascular surgeon. Then, he underwent rehabilitation according to postoperative treatment for gastrocnemius muscle rupture, and finally, he could return to professional boxing matches with victory.

PAES is often initially overlooked by orthopedic surgeons. This is the first report of functional reconstruction surgery and effective rehabilitation performed for PAES. This case focuses on the early diagnosis of and proper orthopedic approach to popliteal artery entrapment syndrome, which is considered important to enable patients to return to high-level sports.

## Introduction

Intermittent claudication in young individuals is first considered a symptom of neurological disorders such as lumbar disc hernia (LDH) or muscular diseases such as medial tibial stress syndrome (MTSS) and chronic exertional compartment syndrome (CECS) in the field of orthopedics [1]. Therefore, peripheral vascular disease is sometimes overlooked. Popliteal artery entrapment syndrome (PAES) is a rare vascular disease observed in young athletes with intermittent claudication.

PAES is classified into six types involving an anomalous relationship between the popliteal artery and its surrounding musculotendinous structures, especially the gastrocnemius muscle [2]. It should be treated promptly when diagnosed to avoid the progression of vascular lesions [1]. In addition, proper surgical treatment and postoperative rehabilitation are important for the return to high-level sports.

Although there have been several reports of PAES [3-5], there are no detailed reports of its surgical treatment and postoperative rehabilitation. Most reports of PAES have been described by cardiovascular surgeons. Therefore, functional reconstruction and rehabilitation have not been focused on until now. Many reports mention surgery with gastrocnemius release only, but gastrocnemius release is not a functional reconstruction for gastrocnemius muscle. Functional reconstruction is necessary for maintaining muscle strength in high-level athletes. Similarly, rehabilitation is essential to return to high-level sports. Rehabilitation of gastrocnemius muscle reconstruction could follow that of gastrocnemius rupture surgery.

We encountered a young professional boxer who was diagnosed with PAES and performed functional reconstruction of the medial gastrocnemius muscle for maintaining muscle strength in collaboration with a cardiovascular surgeon. He also underwent rehabilitation based on postoperative rehabilitation for gastrocnemius muscle rupture. Here, we report this case of PAES and diagnostic methods. In addition, we describe the efficacy of surgical treatment for functional reconstruction and postoperative rehabilitation based on that of gastrocnemius muscle rupture.

## Case presentation

Initial presentation and preoperative evaluation

A 24-year-old male professional boxer with no past medical history and no history of cigarette smoking presented to his orthopedic physician several times because of dull numbness in both his feet during long boxing matches. After the match, his symptoms improved. A year and a half after his first visit to the clinic, his symptoms gradually progressed from numbness to pain in both calves, with the left calf experiencing severe pain. His lower extremity claudication made it difficult for him to continue boxing matches for more than three rounds a month. He was followed up for nearly two years, but the symptoms did not improve. Finally, he was referred to our hospital.

**Figure 1:**
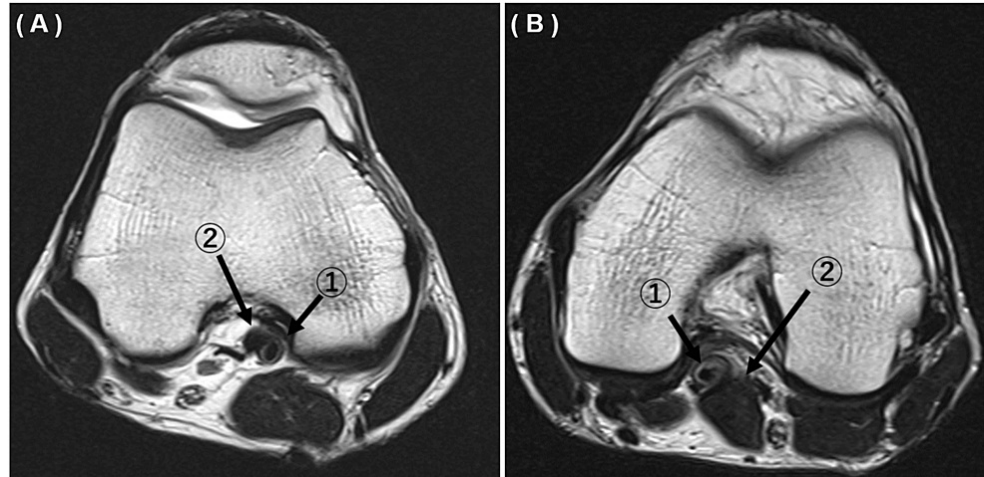
Magnetic resonance imaging of both legs. 1: Popliteal artery. 2: Medial head of the gastrocnemius muscle. A: Right knee. B: Left knee.

Physical examination revealed no limitation to the range of motion (ROM) of both knees, specific discomfort, specific appearance, weakness in the lower legs, neurological findings, or atrophy or hypertrophy. MRI of both knees showed that the medial head of the gastrocnemius muscle was abnormally inclined to the lateral side and the popliteal artery was located on the medial side of the medial head of the gastrocnemius muscle (Figures 1A, 1B). MTSS and CECS were excluded from the differential diagnosis.

Based on these findings, PAES was diagnosed and surgical treatment was performed in collaboration with a cardiovascular surgeon. The patient was fully informed and consented to the publication of this case report.

Intraoperative findings and postoperative rehabilitation

**Figure 2:**
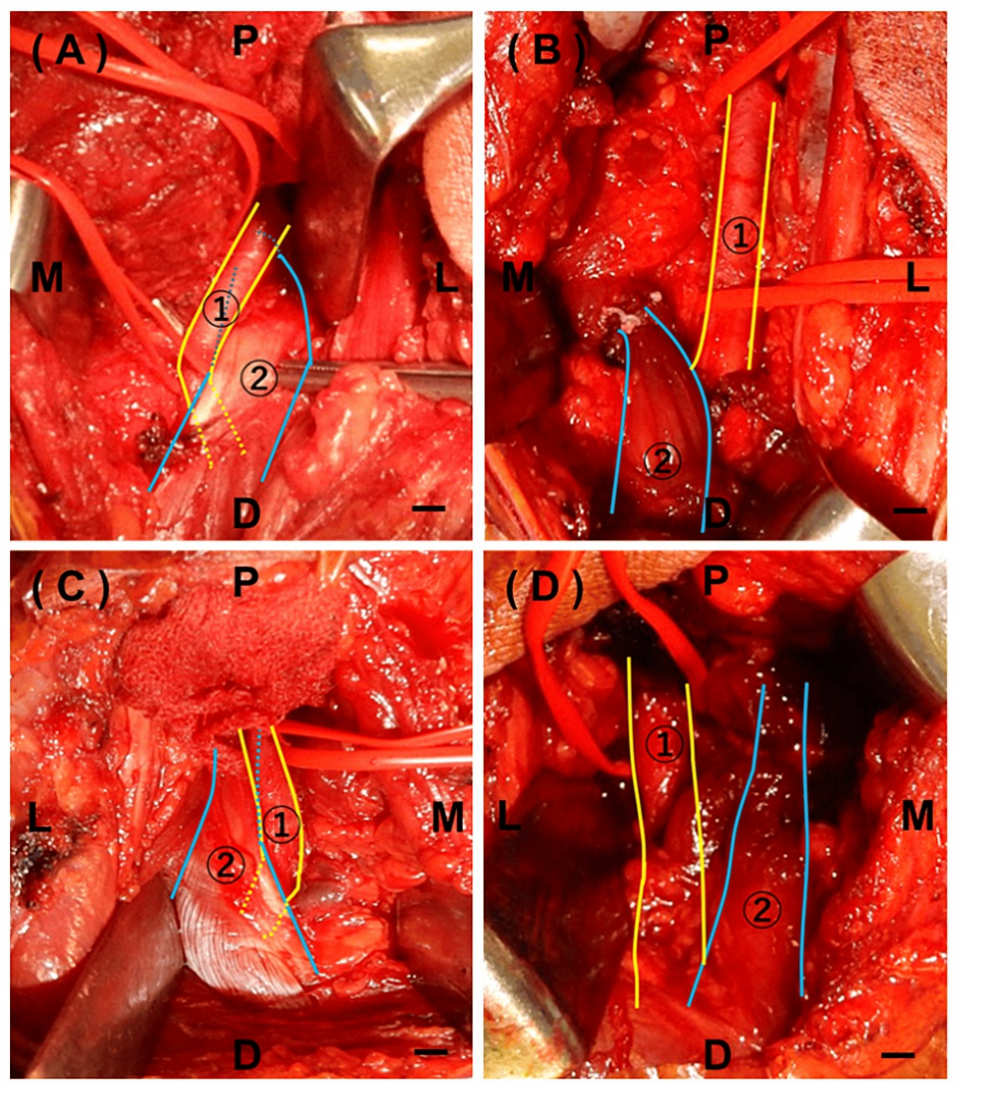
Intraoperative image. P: Proximal. D: Distal. M: Medial. L: Lateral. 1: Popliteal artery. 2: Medial head of the gastrocnemius muscle. Scale bar: 5 mm. A: Before the transition of the medial head of the gastrocnemius muscle of the right knee. B: After the transition of the medial head of the gastrocnemius muscle of the right knee. C: Before the transition of the medial head of the gastrocnemius muscle of the left knee. D: After the transition of the medial head of the gastrocnemius muscle of the left knee.

The patient was placed in the prone position and surgery was performed in the right knee using a posterior S-shaped incision in the popliteal fossa [3,6]. The popliteal artery was compressed medially by the tendon of the medial gastrocnemius muscle (Figure 2A). The medial gastrocnemius head was dissected as proximally as possible to release arterial compression. Subsequently, a suture anchor (Jugger Knot 2.9 mm; Zimmer Biomet, Warsaw, IN, USA) was inserted in the medial posterior condyle of the femur near the normal anatomical attachment on the medial posterior condylar, and the end of the released medial gastrocnemius muscle was fixed using the suture anchor so that the medial head of the gastrocnemius muscle was slightly tense at 20-30° of ankle plantar flexion. We observed that the popliteal artery was running anatomically normal without compression (Figure 2B). We confirmed that the popliteal arteries were not compressed by ankle and knee joint motions. A cardiovascular surgeon used ultrasonography to confirm that the blood flow was good and that no vascular treatment or intervention was required. We performed similar treatment for the left knee (Figures 2C, 2D), and no vascular treatment or intervention was required.

During postoperative rehabilitation, a short splint was fixed to the ankle in plantar flexion at 20° degrees for two weeks after the surgery. We removed the cast after two weeks. The patient was able to start knee ROM exercises within the pain range on the day after surgery. Ankle ROM exercises performed with the knee in flexion were allowed only under the guidance of a physiotherapist. After obtaining sufficient ankle ROM within three weeks after the surgery, the patient was allowed to walk with full weight-bearing gradually. Jogging was permitted at six weeks, and running was permitted at eight weeks after the surgery. At 12 weeks after the surgery, the intermittent claudication had improved, and boxing was permitted. Finally, he could return to professional boxing matches nine months after surgery, and his boxing ranking is rising with his victories.

## Discussion

The most important finding is that functional reconstruction of the medial gastrocnemius muscle and early rehabilitation enabled the patient to return to professional boxing without the preoperative symptoms. So far, PAES has been treated mostly with gastrocnemius release only by cardiovascular surgeons. Moreover, PAES is sometimes overlooked by orthopedic surgeons and should be diagnosed correctly and early.

Anatomical structural abnormality of the popliteal artery was first described in 1879 by a medical student, Anderson Stuart, who noted a variation in its anatomical structure [7]. In 1965, the term “popliteal artery entrapment syndrome” was proposed by Love and Whelan [8]. The incidence of PAES has been reported to range between 0.165% and 3.5% in specific populations [9,10].

PAES is now classified as type I, II, III, IV, V, or VI. The first classification system used for PAES was proposed by Delaney and Gonzales in 1971 [11]. Table 1 shows the six types of PAES [2,5,6,10,11]. The classification is based on the differences in anatomical abnormalities and is divided into types I-IV. Differences in anatomical abnormalities are caused by abnormalities in the developmental process of the popliteal fossa. The developmental processes of the gastrocnemius muscle and popliteal artery occur almost simultaneously. After the medial head of the gastrocnemius muscle crosses the popliteal fossa, new vascular fusion occurs, and the normal anatomical position is established. The popliteal artery is entrapped by the medial head of the gastrocnemius muscle because of the timing of the developmental process and develops into type I, II, or III. The popliteus artery is present on the surface of the popliteus muscle; however, when it remains in the primitive position, deep into the popliteus muscle or fibrous bands, type IV entrapment occurs [10]. In 1979, Rich et al. proposed a type of entrapment mechanism, which compresses both the popliteal vein and artery, as a type V entrapment [6]. Type VI is a functional type of popliteal entrapment with no anatomical anomalies and was proposed by Levien et al. in 1997 [10] (Table 1).

**Table 1:** Types of popliteal artery entrapment syndrome.

Type	Description
Ⅰ	The popliteal artery runs medial to the medial head of the gastrocnemius muscle, inserted in the internal condyle of the femur
Ⅱ	The popliteal artery is compressed medially by the tendon of the medial gastrocnemius muscle that is inserted more lateral than usual
Ⅲ	The popliteal artery is compressed because the gastrocnemius muscle has an additional tendon or fibrous band that inserts laterally
Ⅳ	The popliteal artery remains in a primitive position below the muscle causing its compression, although normal anatomy of the gastrocnemius muscle
Ⅴ	Types I to IV associated with simultaneous popliteal vein compression
Ⅵ	Muscular hypertrophy with normal constitution, resulting in functional popliteal artery and vein entrapment syndrome

Our case was classified as type II and needed surgery to release arterial compression of the medial head of the gastrocnemius muscle. Vascular lesions can progress in PAES patients. Therefore, orthopedists should not misdiagnose PAES in young individuals with intermittent claudication.

For a return to high-activity sports, functional surgical treatment and postoperative rehabilitation should be performed properly with a prompt diagnosis of PAES for maintaining gastrocnemius muscle strength and preventing joint contractures. However, there have been no detailed reports of surgical treatment and postoperative rehabilitation for PAES because most reports have been described by cardiovascular surgeons. This is the first detailed case report of PAES describing diagnostic, surgical, and rehabilitation methods from the perspective of orthopedics.

Diagnostic methods

Diseases that cause intermittent claudication, which are also considered differential diagnoses, include lumbar spinal stenosis, LDH, MTSS, CECS, and peripheral angiopathy (arteriosclerosis obliterans, Burger disease, aneurysm of popliteal artery, etc.). Among them, LDH, MTSS, and CECS present with intermittent claudication in young individuals and athletes [4]. The characteristics of each disease are summarized in Table 2.

**Table 2:** Characteristics of popliteal artery entrapment syndrome (PAES), lumbar disc hernia (LDH), medial tibial stress syndrome (MTSS), and chronic exertional compartment syndrome (CECS).

	PAES	LDH	MTSS	CECS
Age	32	30–40	36.7	<25
Male:Female	9:1~15:1	2–3:1	1:1	1:1
Patient characteristics	Muscular young sportsmen	No particular sport	Novice and recreational runners	Runners, skaters, and military personnel
Area of the lower leg	Back of the calf	Along the innervated area	Distal posteromedial tibia	Commonly in the superficial posterior compartment
Unilateral or bilateral	Bilateral incidence: 30-50%	Commonly unilateral	Commonly unilateral	Bilateral incidence: 50-70%
Imaging	MRI, angiography, and enhanced CT	MRI	Xp, CT, and MRI	MRI and intra-compartmental pressure

The average age of onset is 32 years (range = 20.7-41 years) for PAES. CECS onset occurs at a slightly younger age compared with the other diseases, but no significant difference has been observed [12-14]. Patients with PAES are often muscular, young individuals who play sports; it is also called jogging disease. It develops commonly in males (male:female = 9:1~15:1) [3,9]. The male predominance is a characteristic unlike those of the other differential diseases.

In the early stage, PAES patients have no symptoms or only mild symptoms such as transitory cramps or a feeling of coldness or numbness. In the later stage, if the artery has local stenosis or occlusion, the symptoms are severe ischemia and intermittent claudication. The pain most commonly occurs in the back of the calf; however, it can be anterior and lateral, especially if the anterior tibial artery is involved. The bilateral incidence is relatively high (30-50%). During the physical examination, there may be decreased or absent pulses during forced dorsal ankle flexion or signs of decreased perfusion such as pallor, which is a lower limb thermal gradient. Physical findings are characteristics of PAES compared with other differential diseases [15]. In our case, pulse weakening caused by dorsiflexion was palpable on both sides.

Although the first imaging test is often radiography, it generally shows few findings in young individuals with intermittent claudication. Still, radiography should be performed to rule out any osseous abnormality that may compress an artery [9,16].

In PAES, there is often a variation of pulse and vascular stenosis by flexion of the ankle. Therefore, dynamic Doppler ultrasound may contribute to diagnosis because of the convenience and minimally invasive method. Ultrasound is technically difficult for the diagnosis of PAES, although vascular stenosis and flow can be observed [2,9]. Angiography is also considered one of the most useful methods for diagnosing PAES [2] as the vasculature can be evaluated in detail and the dynamic changes caused by ankle motion can be observed. However, in the case of mild symptoms during the early stages, abnormalities often cannot be found during angiography when performed at rest [5]. MRI or contrast-enhanced CT is desirable for confirmed diagnosis to observe non-anatomical structures between the popliteal artery and the medial gastrocnemius muscle.

Lower limb MRI may be the best method for evaluating the anatomy of the popliteal fossa and may accurately show the structures surrounding the popliteal artery in this region. MRI can also be used to evaluate soft tissue and rule out MTSS [4,5,16] In this case, MRI showed that the left popliteal artery was displaced medially by the medial gastrocnemius at the level of the articular surface and indicated stenosis. Stenosis on the right was also indicated by the medial gastrocnemius head slightly proximal to the articular surface.

Contrast-enhanced CT is useful for performing surgery because it provides accurate anatomy of bone, muscle, and vascular and can also provide three-dimensional images [2,9]. In this case, contrast-enhanced CT confirmed abnormal running of the medial gastrocnemius muscle and the level of vascular stenosis, which indicated the need for surgery. In our view, contrast-enhanced CT may be the most useful method for diagnosing anatomical PAES at any stage.

The physical examination does not always assist with the diagnosis because no clear physical findings appear until after exercise [15]. However, changes in skin tone and palpation of the dorsalis pedis artery in the ankle dorsiflexion position should be evaluated. This finding is important for diagnosing PAES at an early stage. When any abnormal findings are discovered during the physical examination, appropriate imaging tests should be performed.

Surgery

PAES is a progressive disease that requires immediate surgery after diagnosis. When misdiagnosed, patients may experience irreversible arterial stenosis and acute occlusion that could require corrective bypass grafting or thrombectomy [4,5].

The largest case series of PAES included 88 limbs of 48 individuals [1,2,10]. Most reports of surgical treatment describe release only [2]. However, gastrocnemius release alone may cause symptoms similar to gastrocnemius rupture. Rupture of the gastrocnemius is a condition associated with the so-called tennis leg. In the case of a complete rupture, the mass of the shrunk muscle and the gap between the divided ends tend to produce persistent pain and preclude the ability to regain full strength; therefore, reconstruction of the gastrocnemius muscle is desirable [17,18]. In addition, release in PAES is almost always performed at the proximal end of the gastrocnemius muscle, which is equivalent to a proximal tear from the femoral insertion in a gastrocnemius tear. In such cases, weakness of the plantar flexor muscles can occur [19]. There is no evidence that release only is acceptable. Hence, functional reconstruction is considered necessary to avoid muscle weakness and persistent pain. In this case, the patient was a boxer, and surgery was performed because a loss in plantar flexor muscle strength may reduce boxing quality. The dissected tendon was sutured to the anatomical attachment using a suture anchor that could be more firmly fixed to prevent repeat rupture and elongation. The technique with suture anchors is stronger than sewing to soft tissue and easier than the transosseous technique. This operative method can also control appropriate tension and mechanical strength intraoperatively. Release only is easier than reconstruction, but it can lead to muscle weakness.

The gastrocnemius muscle is severely stressed by sudden ankle dorsiflexion during knee extension or sudden knee extension with ankle dorsiflexion [19]. However, if the gastrocnemius muscle is under low tension, functional deterioration occurs. Therefore, surgeons need to confirm the absence of compression in the popliteal artery and fix the released muscle using appropriate tension and mechanical strength by considering the natural plantar flexion of the ankle [19].

Rehabilitation

It has been reported that only about 30% of patients with PAES are able to return to sports, but this may be due to the lack of appropriate rehabilitation [20]. Although there has been no detailed report of rehabilitation after surgery for PAES and no consensus has been reached, rehabilitation should be similar to that provided after surgical treatment of a completely ruptured gastrocnemius muscle [17,19]. As mentioned above, the gastrocnemius muscle is severely stressed by sudden ankle dorsiflexion during knee extension or sudden knee extension with ankle dorsiflexion. To avoid excessive tension, ankle dorsiflexion with knee extension should be avoided in the early days postoperatively [19]. For this reason, rehabilitation after surgical treatment involves a long leg cast for three weeks with knee flexion of 60° and ankle plantar flexion of 20-30° [17]; however, a long leg cast may cause flexion contracture of the knee. Another report used hinged knee braces to prevent knee extension past 30° with flexion limited to 90° [19], but limiting ankle dorsiflexion would not require knee ROM restriction.

In this case, rehabilitation was performed based on treatment after gastrocnemius rupture surgery, as described above [17,18]. As a result, the patient could box within three months and return to professional boxing matches with full ROM and without any symptoms within nine months.

## Conclusions

We performed surgery for bilateral PAES in a young professional boxer with intermittent claudication. He could return to professional boxing after the prompt and appropriate diagnosis, surgery, and rehabilitation. PAES is often initially overlooked by orthopedic surgeons, although it is a progressive disease, with late diagnosis often leading to severe artery lesions. Most reports of PAES have been described by cardiovascular surgeons. Therefore, functional reconstruction and rehabilitation have not been mentioned enough until now. This is the first detailed report of functional reconstruction and rehabilitation performed for PAES. In this case, functional reconstruction of the gastrocnemius muscle and early rehabilitation based on postoperative rehabilitation for gastrocnemius muscle rupture were performed, and the patient could return to playing high-level sports as a professional boxer. It is suggested that early diagnosis of and proper orthopedic approach for PAES is efficient in enabling patients to return to high-level sports.

## References

[1] Gokkus K, Sagtas E, Bakalim T, Taskaya E, Aydin AT (2014). Popliteal entrapment syndrome. A systematic review of the literature and case presentation. Muscles Ligaments Tendons J.

[2] Sinha S, Houghton J, Holt PJ, Thompson MM, Loftus IM, Hinchliffe RJ (2012). Popliteal entrapment syndrome. J Vasc Surg.

[3] O'Leary DP, O'Brien G, Fulton G (2010). Popliteal artery entrapment syndrome. Int J Surg Case Rep.

[4] Koehler RM, Cimbak NC, Parisien RL, Nicoletta RJ, Kalish JA (2020). Bilateral popliteal artery entrapment syndrome in a young female NCAA Division-I collegiate basketball player: a case report. JBJS Case Connect.

[5] Carneiro Júnior FC, Carrijo EN, Araújo ST, Nakano LC, de Amorim JE, Cacione DG (2018). Popliteal artery entrapment syndrome: a case report and review of the literature. Am J Case Rep.

[6] Rich NM, Collins GJ Jr, McDonald PT, Kozloff L, Clagett GP, Collins JT (1979). Popliteal vascular entrapment. Its increasing interest. Arch Surg.

[7] Stuart TP (1879). Note on a variation in the course of the popliteal artery. J Anat Physiol.

[8] Love JW, Whelan TJ (1965). Popliteal artery entrapment syndrome. Am J Surg.

[9] Grimm NL, Danilkowicz R, Shortell C, Toth AP (2020). Popliteal artery entrapment syndrome. JBJS Rev.

[10] Levien LJ, Veller MG (1999). Popliteal artery entrapment syndrome: more common than previously recognized. J Vasc Surg.

[11] Delaney TA, Gonzalez LL (1971). Occlusion of popliteal artery due to muscular entrapment. Surgery.

[12] Wilson CA, Roffey DM, Chow D, Alkherayf F, Wai EK (2016). A systematic review of preoperative predictors for postoperative clinical outcomes following lumbar discectomy. Spine J.

[13] Menéndez C, Batalla L, Prieto A, Rodríguez MÁ, Crespo I, Olmedillas H (2020). Medial tibial stress syndrome in novice and recreational runners: a systematic review. Int J Environ Res Public Health.

[14] Velasco TO, Leggit JC (2020). Chronic exertional compartment syndrome: a clinical update. Curr Sports Med Rep.

[15] Pham TT, Kapur R, Harwood MI (2007). Exertional leg pain: teasing out arterial entrapments. Curr Sports Med Rep.

[16] Pell RF 4th, Khanuja HS, Cooley GR (2004). Leg pain in the running athlete. J Am Acad Orthop Surg.

[17] Cheng Y, Yang HL, Sun ZY, Ni L, Zhang HT (2012). Surgical treatment of gastrocnemius muscle ruptures. Orthop Surg.

[18] Pereira VL, Andreoli CV, Santos RF, Belangero PS, Ejnisman B, de Castro Pochini A (2022). Surgical repair of the medial head of the gastrocnemius: two case reports and review. J Surg Case Rep.

[19] Cooper J, Arner JW, Peebles LA, Provencher MT (2021). Surgical treatment of medial gastrocnemius tear. Arthrosc Tech.

[20] Deveze E, Bruneau A, Hersant J, Ammi M, Abraham P, Picquet J (2023). Popliteal entrapment syndrome: diagnostic, surgical management, and short-term results of a ten-year experience. Ann Vasc Surg.

